# HIF-2α mediates hypoxia-induced LIF expression in human colorectal cancer cells

**DOI:** 10.18632/oncotarget.3017

**Published:** 2015-01-22

**Authors:** Lihua Wu, Haiyang Yu, Yuhan Zhao, Cen Zhang, Jiabei Wang, Xuetian Yue, Qifeng Yang, Wenwei Hu

**Affiliations:** ^1^ Rutgers Cancer Institute of New Jersey, Rutgers the State University of New Jersey, New Brunswick, NJ, USA; ^2^ First Affiliated Hospital, Zhejiang University, Hangzhou, China; ^3^ Department of Breast Surgery, Qilu Hospital, Shandong University, Jinan, China

**Keywords:** Leukemia inhibitory factor, hypoxia, HIF-2α, hypoxia-responsive element

## Abstract

Leukemia inhibitory factor (LIF), a multi-functional cytokine, has a complex role in cancer. While LIF induces the differentiation of several myeloid leukemia cells and inhibits their growth, it also promotes tumor progression, metastasis and chemoresistance in many solid tumors. LIF is frequently overexpressed in a variety of human tumors and its overexpression is often associated with poor prognosis of patients. Currently, the mechanism for LIF overexpression in tumor cells is not well-understood. Here, we report that hypoxia, a hallmark of solid tumors, induced LIF mRNA expression in human colorectal cancer cells. Analysis of LIF promoter revealed several hypoxia-responsive elements (HREs) that can specifically interact with and be transactivated by HIF-2α but not HIF-1α. Consistently, ectopic expression of HIF-2α but not HIF-1α transcriptionally induced LIF expression levels in cells. Knockdown of endogenous HIF-2α but not HIF-1α by siRNA largely abolished the induction of LIF by hypoxia in cells. Furthermore, there is a strong association of HIF-2α overexpression with LIF overexpression in human colorectal cancer specimens. In summary, results from this study demonstrate that hypoxia induces LIF expression in human cancer cells mainly through HIF-2α, which could be an important underlying mechanism for LIF overexpression in human cancers.

## INTRODUCTION

Leukemia inhibitory factor (LIF), a member of the interleukin-6 cytokine superfamily, is a pleiotropic protein expressed in multiple types of tissues and cells which regulates an array of important biological functions. For example, LIF maintains the pluripotency of embryonic stem cells, while induces the differentiation of several myeloid leukemia cells and inhibits their growth [1, 2]. The study from LIF knock-out mice revealed that LIF is essential for the implantation of blastocysts [3]. Our recent study showed that LIF is a p53 target gene which mediates the function of p53 in implantation [4, 5]. LIF functions through binding to its receptor complex composed of LIF receptor (LIF-R) and glycoprotein gp130 to selectively activate different signaling pathways, including Jak/Stat3, ERK/MAPK, PI3K/AKT and mTOR pathways, depending upon different context of cells and tissues [6–8].

LIF plays an important and complex role in cancer depending upon the types of the cancer. While early studies showed that LIF induces the differentiation of myeloid leukemia cells, recent studies from others and our laboratory demonstrated that LIF can inhibit cell differentiation and promote proliferation of many types of tumor cell lines [9–11]. LIF has been reported to promote the progression of malignancies of many solid tumors, including rhabdomyosarcoma, choriocarcinoma, melanoma, breast cancer and colorectal cancer [8, 10, 12–17]. While the mechanisms of the promoting effect of LIF on the development and progression of solid tumors are not well-understood, our recent study showed that LIF is a novel negative regulator of p53 through the Stat3/ID1/MDM2 signaling in human colorectal cancers [17]. LIF can activate the Stat3 signaling to transcriptionally induce ID1, which in turn increases the levels of MDM2, a key negative regulator of p53 to down-regulate p53 protein levels and function. Overexpression of LIF promotes chemo-resistance in human colorectal cancers through attenuating p53 levels and functions in cells [17].

LIF is frequently overexpressed in a variety of solid tumors including colorectal cancers, breast cancers and skin cancers [8, 14, 17]. Importantly, the overexpression of LIF in tumors often correlates with poor prognosis of patients [8, 17, 18]. However, the mechanism for LIF overexpression in tumors is not well-understood. Hypoxia is a hallmark of solid tumors, including colorectal cancers [19]. In this study, we studied the effect of hypoxia on LIF expression in colorectal cancer cells. We found that LIF is transcriptionally induced by hypoxia in human colorectal cancer cell lines. Furthermore, the induction of LIF expression by hypoxia is mainly mediated by HIF-2α. Indeed, there is a strong association of HIF-2α overexpression with LIF overexpression in human colorectal cancer specimens (*n* = 284). Results from this study clearly demonstrate that hypoxia induces LIF expression mainly through HIF-2α as an important underlying mechanism for LIF overexpression in human colorectal cancers.

## RESULTS

### The induction of LIF expression by hypoxia in human colorectal cancer cells

**Figure 1:**
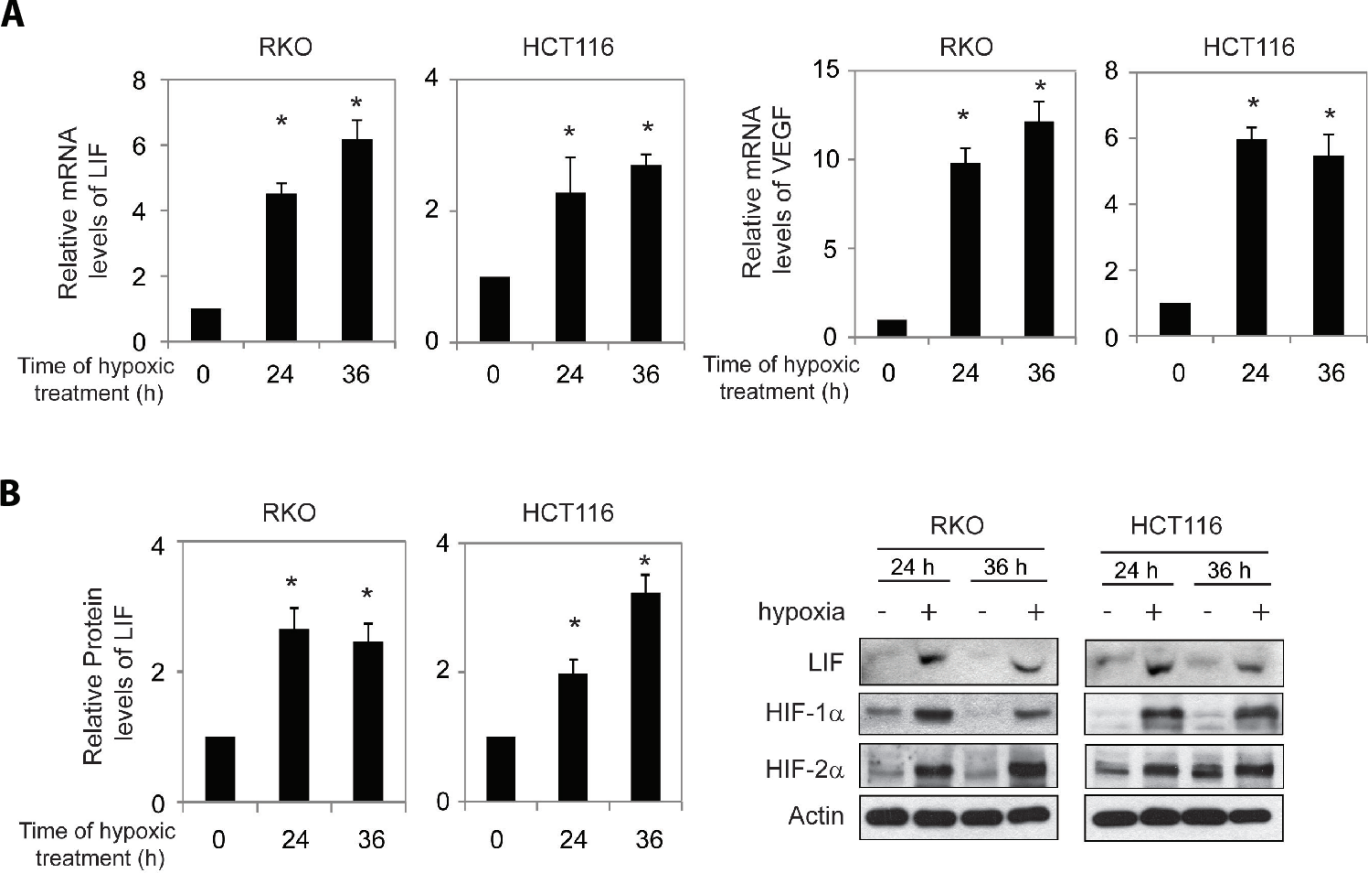
Hypoxia induces LIF expression levels in human colorectal cancer cell lines Human colorectal cancer cell lines RKO and HCT116 cells were cultured under the hypoxic condition for the indicated time periods. **(A)** The mRNA expression levels of LIF in these cells were determined by Taqman real-time PCR and normalized with actin (left panels). The mRNA expression levels of VEGF in these cells were determined as a positive control (right panels). **(B)** The LIF protein levels were determined by Elisa assays (left panels) and Western-blot assays (right panels), respectively. Data are presented as mean ± SD (*n* = 3). *:*p* < 0.01, Student's *t*-test.

Results from our recent study have shown that a high percentage of human colorectal cancer specimens display elevated LIF expression levels [17]. Furthermore, higher levels of LIF in colorectal cancers are associated with poor prognosis of colorectal cancer patients [17]. Currently, the underlying mechanism for the increased expression of LIF in colorectal cancers is unclear. Gene amplification is a common event leading to the overexpression of some p53 negative regulators in tumors, such as MDM2 and MDM4 [20, 21]. Employing the cBio Cancer Genomics Portal (http://cbioportal.org), the copy number variation of the *LIF* gene was analyzed in colorectal adenocarcinoma triplets MSKCC database (*n* = 138) and colorectal adenocarcinoma TCGA provisional database (*n* = 601) [22]. No amplification of the *LIF* gene was found in both databases (Table 1). This result suggests that LIF overexpression in colorectal cancer is not mainly contributed by gene amplification. Hypoxia is a hallmark of solid tumors, including colorectal cancers [19]. Interestingly, we found that LIF expression was induced by hypoxia. Human colorectal cancer cell lines RKO and HCT116 cells were subjected to hypoxia treatment (0.1% O_2_). The effective induction of hypoxia in cells was confirmed by the increased expression of VEGF, a hypoxia-responsive gene, at mRNA levels (Figure 1A) and the increased protein levels of HIF-1α and HIF-2α, two major hypoxia inducible factors (HIFs) (Figure 1B). Notably, LIF mRNA levels were significantly increased (*p* < 0.01) in both RKO and HCT116 cells cultured under the hypoxic condition as determined by Taqman real-time PCR (Figure 1A). The induction of LIF expression by hypoxia was confirmed at the protein levels in these cells by using Elisa assays and Western-blot assays, respectively (Figure 1B). These data clearly demonstrated that hypoxia induces LIF expression in human colorectal cancer cells.

**Table 1:** No amplification of the *LIF* gene was detected in human colorectal cancer specimens

Colorectal Adenocarcinoma Triplets (MSKCC)	
Number of cases with LIF amplification	0
Total number of cases	138
Colorectal Adenocarcinoma (TCGA, Provisional)	
Number of cases with LIF amplification	0
Total number of cases	601

The information of amplification of the *LIF* gene in both databases was obtained from the cBio Cancer Genomics Portal (http://cbioportal.org).

### Hypoxia transactivates the *LIF* promoter region containing hypoxia-responsive elements (HREs)

**Figure 2:**
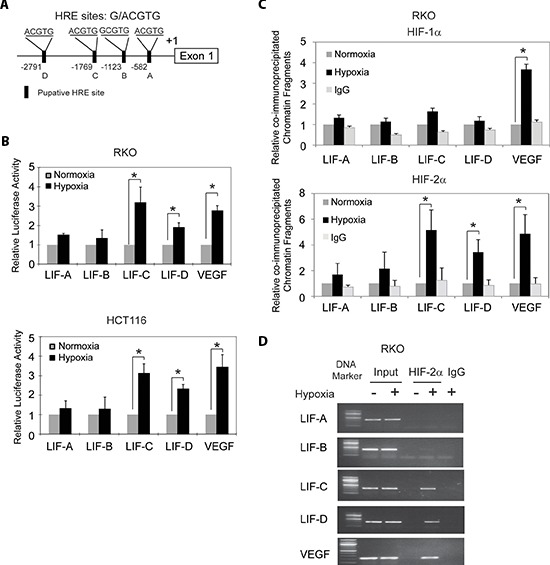
Hypoxia transactivates hypoxia-responsive elements (HREs) in the *LIF* promoter through HIF-2α **(A)** The human *LIF* gene contains 4 putative HREs in its promoter region. **(B)** Hypoxia activates the luciferase activity of reporter vectors containing HRE-C or HRE-D sites in the *LIF* promoter. RKO and HCT116 cells were transfected with the luciferase reporter vectors, and then subjected to hypoxia treatment for 36 h before measuring luciferase activities. Luciferase reporter vectors containing the HRE site in the *VEGF* promoter was included as a positive control. **(C)** and **(D)** HIF-2α but not HIF-1α binds to HRE-C and HRE-D sites in the *LIF* promoter under the hypoxic condition in RKO cells as determined by ChIP assays. Cells were cultured under the hypoxic or normoxic conditions for 36 h before assays. The HRE site in the *VEGF* promoter serves as a positive control. The amount of DNA fragments pulled-down was determined by real-time PCR **(C)** or conventional PCR **(D)**. Data are presented as mean ± SD (*n* = 3). *:*p* < 0.01 (Student's *t*-test).

The transcriptional response to hypoxia in cells is largely mediated by HIFs. HIFs are heterodimeric transcription factors that are composed of an α-subunit and a β-subunit of helix-loop-helix-PAS family proteins. HIFs bind to DNA containing a hypoxia-responsive element (HRE; 5′-G/ACGTG-3′) [23]. Under the hypoxic condition, HIFs are stabilized and the transcriptional activities of HIFs increase. HIF-1α and HIF-2α are two major α-subunits of HIFs. HIF-1α and HIF-2α induce a number of common genes, and at the same time they each have their unique target genes [23]. To investigate whether the transcriptional induction of LIF by hypoxia is mediated by HIFs, we searched for the HRE consensus sequence in the promoter region of the *LIF* gene from 3 kb upstream of transcriptional site to exon 1. Four putative HRE sites (HRE-A, HRE-B, HRE-C and HRE-D) were identified in the promoter region of the *LIF* gene (Figure 2A).

To investigate whether these putative HREs account for the hypoxia-mediated induction of LIF, the DNA fragments containing one copy of these putative HRE sites were inserted into a pGL2 luciferase reporter plasmid. *VEGF* is a well-known hypoxia-inducible gene. HIFs physically interact with the HRE in the *VEGF* promoter and induce VEGF expression levels under hypoxia [24]. The DNA fragment containing the HRE in the *VEGF* promoter was inserted into a pGL2 luciferase reporter plasmid to serve as a positive control. RKO and HCT116 cells were transiently transfected with these reporter constructs, and then exposed to hypoxia for 36 h. pRL-SV40-TK plasmids were co-transfected as an internal standard to normalize transfection efficiency. As shown in Figure 2B, hypoxia clearly enhanced luciferase expression levels of the reporter plasmids containing the HRE from the *VEGF* promoter, the HRE-C and HRE-D from the *LIF* promoter, but did not have a clear effect on HRE-A or HRE-B form the *LIF* promoter in both cells. These results indicate that hypoxia transactivates the *LIF* promoter containing functional HREs (HRE-C and HRE-D).

### HIF-2α instead of HIF-1α binds to HRE-C and HRE-D in the *LIF* promoter under the hypoxic condition

To further investigate which HIF (HIF-1α or HIF-2α) mediates the hypoxia-induced LIF expression, chromatin immunoprecipitation (ChIP) assays were employed to determine whether HIF-1α and HIF-2α physically bind to HREs, especially HRE-C and HRE-D in the *LIF* promoter. RKO cells were cultured under the normoxic or hypoxic conditions for 36 h, and the ChIP assays were performed using an antibody against HIF-1α or HIF-2α. The degree of pull-down of chromatin fragments was determined by quantitative real-time PCR. As shown in Figure 2C, both HIF-1α and HIF-2α interacted with the chromatin fragments containing the HRE from the *VEGF* promoter under the hypoxic but not normoxic conditions. However, no clear immunoprecipitation of the chromatin fragments containing HRE-A, HRE-B, HRE-C and HRE-D by the antibody against HIF-1α was observed in RKO cells under either the hypoxic or normoxic conditions (Figure 2C). Interestingly, the chromatin fragments containing HRE-C and HRE-D but not HRE-A and HRE-B were pulled down by the antibody against HIF-2α in RKO cells under the hypoxic condition (Figure 2C). Furthermore, these chromatin fragments were not co-immunoprecipitated with the HIF-2α antibody in cells under the normoxic condition (Figure 2C). The specific pull-down of chromatin fragment containing HRE-C and HRE-D but not HRE-A and HRE-B by the HIF-2α antibody under the hypoxic condition was also confirmed by conventional PCR followed by agarose gel electrophoresis (Figure 2D). These results demonstrated that HIF-2α but not HIF-1α interacts with HRE-C and HRE-D in the *LIF* promoter under the hypoxic condition, which may mediate the hypoxia-induced LIF expression.

### HIF-2α transcriptionally regulates LIF expression

**Figure 3:**
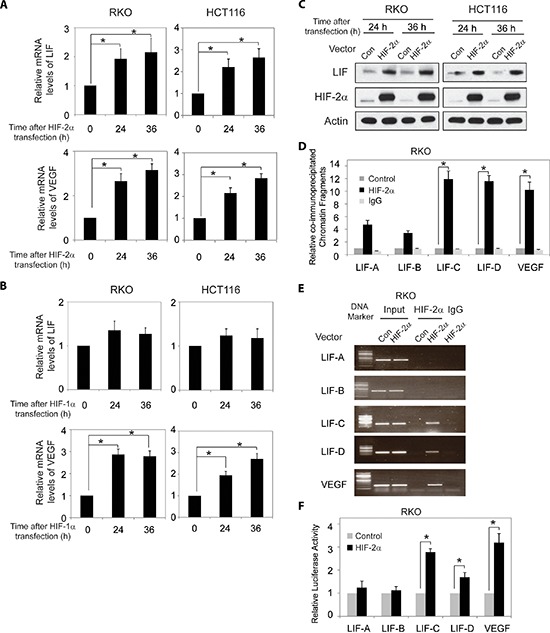
HIF-2α transcriptionally regulates LIF expression **(A)** Ectopic HIF-2α expression increases LIF and VEGF mRNA expression levels in RKO and HCT116 cells. The mRNA expression levels of LIF and VEGF were determined by Taqman real-time PCR and normalized with actin. **(B)** Ectopic HIF-1α expression increases VEGF mRNA expression levels but has no obvious effect on LIF mRNA expression levels in RKO and HCT116 cells. **(C)** Ectopic HIF-2α expression increases LIF protein levels in RKO and HCT116 cells as determined by Western-blot assays. **(D)** and **(E)** HIF-2α binds to HRE-C and HRE-D sites in the *LIF* promoter in RKO cells transfected with HIF-2α expression plasmids as determined by ChIP assays. The amount of DNA fragments pulled-down was determined by real-time PCR **(D)** or conventional PCR **(E)**. The HRE site in the *VEGF* promoter serves as a positive control. **(F)** HIF-2α activates luciferase activity of reporter vectors containing HRE-C or HRE-D sites in the *LIF* promoter in RKO cells transfected with HIF-2α expression plasmids. Luciferase reporter vectors containing the HRE site in the *VEGF* promoter was included as a positive control. Data are presented as mean ± SD (*n* = 3). *:*p* < 0.01 (Student's *t*-test).

To directly investigate whether HIF-2α but not HIF-1α transcriptionally induces LIF expression levels, RKO and HCT116 cells were transfected with the plasmids expressing HIF-2α (pcDNA3-HA-HIF2α) and HIF-1α (pcDNA3-HA-HIF1α), respectively. The ectopic expression of either HIF-1α or HIF-2α clearly induced VEGF mRNA expression (Figure 3A and 3B). Ectopic HIF-2α expression clearly increased LIF expression at both mRNA and protein levels as determined by Taqman Real-time PCR and Western-blot assays, respectively (Figure 3A and 3C). The ectopic HIF-2α expression in cells was confirmed at the protein level by Western-blot assays (Figure 3C). However, ectopic HIF-1α expression had no apparent effect on LIF expression as determined at the mRNA level by Taqman Real-time PCR assays (Figure 3B).

Furthermore, the degree of pull-down of chromatin fragments containing HREs in the *LIF* promoter by HIF-2α was determined by ChIP assays in RKO cells with ectopic HIF-2α expression. As shown in Figure 3D and 3E, chromatin fragments containing HRE-C and HRE-D but not HRE-A and HRE-B were co-immunoprecipitated with the HIF-2α antibody in cells with ectopic HIF-2α expression as determined by both quantitative real-time PCR and conventional PCR assays. These observations clearly showed that HIF-2α interacted with HRE-C and HRE-D in the *LIF* promoter, which is consistent with the ChIP results obtained in cells under the hypoxic condition.

We further investigated whether HIF-2α directly transactivates HREs in the *LIF* promoter. RKO cells were co-transfected with an expression plasmid of HIF-2α and the pGL2 reporter plasmids containing HREs in the *LIF* promoter or the HRE in the *VEGF* promoter along with pRL-SV40-TK plasmids. Ectopic HIF-2α expression increased the luciferase activity of the reporter plasmids containing the HRE in the *VEGF* promoter (Figure 3F), which is consistent with a previous report [25]. Notably, ectopic HIF-2α expression clearly transactivated the reporter plasmids containing HRE-C and HRE-D from the *LIF* promoter, but had a minimal effect on the reporter plasmids containing HRE-A and B from the *LIF* promoter (Figure 3F). Collectively, these results clearly showed that HIF-2α interacts with and transactivates HRE-C and HRE-D in the *LIF* promoter to directly induce LIF expression in cells.

### HIF-2α mediates the induction of LIF expression by hypoxia

**Figure 4:**
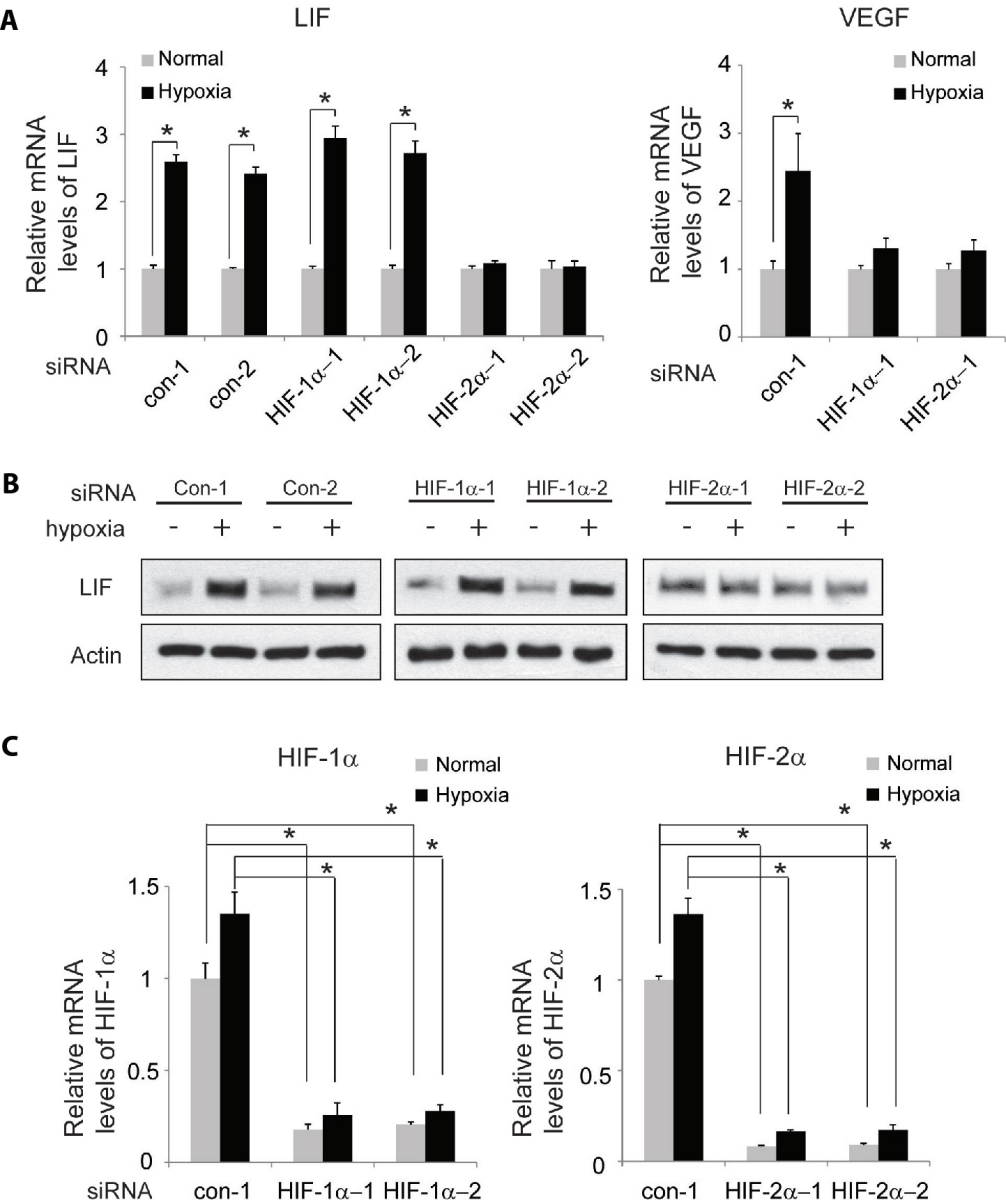
HIF-2α mediates the induction of LIF expression by hypoxia Knockdown of endogenous HIF-2α but not HIF-1α largely abolishes the induction of LIF expression by hypoxia in RKO cells. Cells with knockdown of endogenous HIF-1α, HIF-2α by siRNA oligos or transfected with control siRNA were treated with hypoxia for 36 h. Two different siRNA oligos against HIF-1α and HIF-2α, respectively, were used, and similar results were obtained. **(A)** The mRNA expression levels of LIF (left panel) and VEGF (right panel) were determined by Taqman real-time PCR and normalized with actin. **(B)** The LIF protein levels were determined by Western-blot assays. **(C)** The knockdown of HIF-1α (left panel) and HIF-2α (right panel) in cells was confirmed at the mRNA level by Taqman real-time PCR and normalized with actin. Data are presented as mean ± SD (*n* = 3). *:*p* < 0.01 (Student's *t*-test).

To investigate whether HIF-2α but not HIF-1α mediates the induction of LIF expression by hypoxia, the effect of hypoxia on LIF expression levels was determined in RKO cells with knock-down of endogenous HIF-1α or HIF-2α by siRNA. As shown in Figure 4A and 4B, knock-down of endogenous HIF-2α largely abolished hypoxia-induced LIF expression in RKO cells as determined at both mRNA and protein levels by Taqman real-time PCR and Western-blot assays, respectively. In contrary, knock-down of endogenous HIF-1α by siRNA had no apparent effect on hypoxia-induced LIF expression (Figure 4A and 4B). It has been shown that VEGF is a common target of HIF-1α and HIF-2α. Indeed, knock-down of either HIF-1α or HIF-2α can all greatly inhibit hypoxia-induced VEGF expression (Figure 4A). The effective knock-down of HIF-1α and HIF-2α was confirmed at the mRNA level by Taqman real-time PCR assays (Figure 4C). These results clearly demonstrated that hypoxia induces LIF expression mainly through HIF-2α.

### HIF-2α overexpression is associated with LIF overexpression in human colorectal cancer specimens

**Figure 5:**
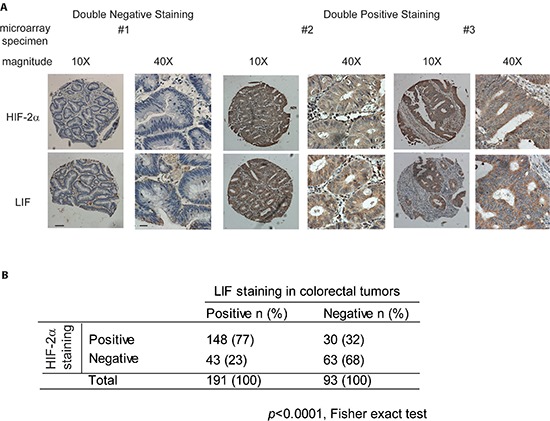
HIF-2α overexpression is associated with LIF overexpression in human colorectal cancer specimens HIF-2α and LIF protein levels were determined by IHC staining in tissue microarrays containing 284 cases of human colorectal cancer specimens. **(A)** Representative IHC staining results for HIF-2α (upper panels) and LIF (lower panels) are shown. Positive HIF-2α or LIF staining: > 10% cells stained with HIF-2α or LIF, respectively. Scale bar: 50 μm for low magnitude images (10X); 10 μm for high magnitude images (40X). **(B)** HIF-2α overexpression is associated with LIF overexpression in human colorectal tumors (*p* < 0.0001, Fisher exact test).

In a recent study, we reported that LIF is frequently overexpressed in human colorectal cancer samples [17]. In that study, we found that a significantly higher percentage of colorectal cancer samples showed positive LIF staining (> 10% cells are stained) compared with their matched adjacent normal tissues (72% *vs*. 20%, *p* < 0.005) as determined by IHC staining in formalin-fixed and paraffin-embedded (FFPE) colorectal cancer samples and their matched adjacent normal tissues [17]. To investigate whether hypoxia and HIF-2α contribute to the increased expression of LIF in human colorectal cancer samples, the expression levels of LIF and HIF-2α were determined in the tissue microarray comprising duplicate paraffin tissue cores of 284 human colorectal tumor samples by IHC staining. The representative IHC images of LIF and HIF-2α were shown in Figure 5A. Consistent with our previous finding [17], a significant portion of colorectal cancer samples (67%; 191 out of 284 cases) showed positive LIF staining. The overexpression of LIF was observed in all stages of colorectal cancer samples (82% in stage I, 18 of 22 cases; 64% in stage II, 148 of 232 cases; 86% in stage III, 19 of 22 cases; and 75% in stage IV, 6 of 8 cases). There is also a significant portion of colorectal cancer samples (64%; 183 out of 284 cases) showed positive HIF-2α staining (> 10% cells are stained). The percentage of colorectal cancer samples with positive HIF-2α staining is much higher in late stages (stages III & IV) than in early stages (stages I & II) (45% in stage I, 10 of 22 cases; 63% in stage II, 146 of 232 cases; 91% in stage III, 20 of 22 cases; and 88% in stage IV, 7 of 8 cases). Notably, HIF-2α overexpression is strongly associated with LIF overexpression (Figure 5B) (*p* < 0.0001, Fisher exact test). HIF-2α positive staining was observed in 77% of cases with LIF positive staining (148 of 191 cases), but only in 32% of cases with LIF negative staining (30 of 93 cases). These results strongly suggested that the transcriptional induction of LIF by hypoxia and HIF-2α is an important mechanism accounting for the LIF overexpression in human colorectal cancers.

## DISCUSSION

LIF is a multi-functional cytokine. LIF has critical roles in blastocyst implantation process, immune response to inflammation and maintaining pluripotency of embryonic stem cell [1, 26]. Recent studies including ours showed that LIF promotes tumor development and progression in many solid tumors through multiple mechanisms. For example, results from our recent study showed that overexpression of LIF promotes growth and metastasis of breast cancer cells both *in vitro* and *in vivo* through the activation of the AKT-mTOR pathway [8]. A very recent study reported that the induction of LIF expression in cancer-associated fibroblasts can promote onset of a proinvasive microenvironment [27]. In addition to breast cancer, the promoting effect of LIF on metastasis has also been observed in rhabdomyosarcoma and melanoma [13, 14, 17]. Our recent study showed that LIF down-regulates p53 protein levels and function, which in turn promotes chemo-resistance in human colorectal cancer [17]. Collectively, these reports and findings strongly suggest the important role of LIF in tumor development and progression.

LIF is frequently overexpressed in many solid tumors, including breast cancer, colorectal cancers and nasopharyngeal cancers, and the overexpression of LIF in tumors is often associated with poor prognosis of patients [8, 17, 18]. The mechanisms for LIF overexpression in tumors are not well-understood. Our previous work demonstrated that LIF is a transcriptional target of p53; the expression of LIF in uterine tissue is under the control of the p53 pathway to ensure the proper implantation process [4, 5, 28]. However, since majority of cancers have dysfunctional p53 signaling [29–31], LIF overexpression in a significant portion of human tumors could not be explained by the regulation of LIF by p53. Gene amplification is a common event leading to the overexpression of some p53 negative regulators in tumors, such as MDM2 and MDM4 [20, 21]. We analyzed the copy number of the *LIF* gene in over 700 cases of human colorectal cancer samples in cBio Cancer Genomics Portal, and did not find *LIF* amplification.

In this study, we provided the evidence that hypoxia, a hallmark of solid tumors, induces the expression of LIF in human colorectal cancer cell lines. The transcriptional induction of LIF by hypoxia is mainly mediated by HIF-2α. Ectopic HIF-2α expression transcriptionally induces the expression of LIF through binding to and transactivating HRE sites in the *LIF* promoter. Knock-down of endogenous HIF-2α largely abolishes the induction of LIF by hypoxia. Furthermore, there is a strong association of HIF-2α overexpression with LIF overexpression in human colorectal cancer specimens (*n* = 284, *p* < 0.0001).

HIF-1α and HIF-2α are important hypoxia inducible proteins that mediate majority of hypoxic responses in cells. Both HIF-1α and HIF-2α proteins are often overexpressed in a variety of human tumors, especially solid tumors, and tumor-derived cell lines [32]. HIF-1α and HIF-2α proteins can form dimers with HIF-β and bind to HRE to effectively regulate the expression levels of their target genes. While HIF-1α and HIF-2α proteins share some similar properties mainly though transcriptional regulation of a group of common target genes, they each have their unique target genes which contribute to the unique characteristics of these two proteins. HIF-1α and HIF-2α proteins have two transactivation domains: N-terminal transactivation domain (N-TAD) and 2^nd^ transactivation domain (C-TAD). The structure of N-TAD is different between two proteins, which has been suggested to contribute to the target gene specificity of HIF-1α and HIF-2α. The C-TAD is highly homologous between two proteins, and has been suggested to contribute to the transcriptional regulation of their common target genes [33]. In addition, the differences of HIF-1α and HIF-2α target gene selection can be contributed by their interactions with different transcription factors and/or chromatin context [33, 34]. It has been reported that HIF-1α preferentially induces genes in the glycolytic pathway and HIF-2α is involved in the regulation of genes important for tumor growth, cell cycle progression and maintaining stem cell pluripotency, including c-Myc and the stem cell factor OCT-3/4 [35–38].

In this study, we found that LIF is a target gene of HIF-2α but not HIF-1α. The *LIF* promoter contains 4 putative HRE sites (HRE-A, HRE-B, HRE-C and HRE-D). Results from the ChIP assays showed that HIF-2α but not HIF-1α interacted with HRE-C and HRE-D in the *LIF* promoter under the hypoxic condition. The underlying mechanism for the selective binding of HIF-2α towards HRE-C and HRE-D but not HRE-A and HRE-B is currently unclear. It is possible that this selectivity is contributed by flanking sequences and/or chromatin context of these HRE sites. Knock-down of HIF-2α but not HIF-1α largely abolished hypoxia-induced LIF expression although knock-down of either HIF-1α or HIF-2α can greatly inhibit hypoxa-induced VEGF expression. These results clearly demonstrate that while VEGF is a common target gene for both HIF-1α and HIF-2α, LIF is a target gene for HIF-2α but not HIF-1α. LIF is predominantly induced under the hypoxic condition through HIF-2α but not HIF-1α. It is worth noting that under chronic hypoxic condition, HIF-1α proteins are degraded over time, whereas HIF-2α proteins are often continuously accumulated [39, 40]. It is therefore suggested that HIF-1α primarily mediates the fast (acute) response to hypoxia, and HIF-2α mediates the late (chronic) response to hypoxia [39]. The continuous accumulation of HIF-2α under the hypoxic condition will lead to continuous induction of LIF in solid tumors with chronic hypoxic condition, which in turn can promote tumor development and progression. Consistently, a previous study on the association of the expression levels of HIF-1α and HIF-2α with clinicopathological factors in human colorectal cancers showed that most of the clinicopahtological factors representing the tumor aggressiveness were significantly correlated with overexpression of HIF-2α but not HIF-1α [41].

In summary, the results from this study demonstrated that LIF expression is stimulated by hypoxia in human colorectal cancer cells. LIF induction under hypoxia is predominantly mediated by HIF-2α. Considering the critical role of LIF in promoting tumor development and chemo-resistance in tumors, the specific regulation of LIF expression by HIF-2α provides foundation for future study to develop potential therapeutic strategies to block LIF overexpression in tumors.

## METHODS

### Cell culture and cell treatment

Human colorectal cancer cell lines RKO and HCT116 cells were obtained from ATCC. Cells were maintained in DMEM supplemented with 10% fetal bovine serum (Sigma). For hypoxic treatment, cells at 50–60% confluence were incubated in a hypoxia chamber (0.1% O_2_, Billups-Rothenberg). HIF-2α expression plasmids (pcDNA3-HA-HIF-2α) were obtained from Addgene. HIF-1α expression plasmids (pcDNA3-HA-HIF-1α) were kindly provided by Dr. L.E. Huang (University of Utah, Salt Lake, UT). For siRNA knockdown, two different siRNA oligos against HIF-1α and HIF-2α were purchased from IDT. siRNA targeting HIF-1α: siRNA-1: 5′ CAGACUUUAUGUUCAUAGUUCUUCCUC 3′; siRNA-2: 5′ CUUCCACAACUACAUAGGGUAUUG UUU 3′. siRNA targeting HIF-2α: siRNA-1: 5′ AUACA GUUAUAAUGUUGUCAGUAGGAA 3′; siRNA-2: 5′ UCAUUGAAAUCCGUCUGGGUACUGCAU 3′. Expression plasmids and siRNA oligos were transfected into cells using Lipofectamine 2000 (Invitrogen).

### Taqman real-time PCR

Total RNA was isolated by using RNeasy Kit (Qiagen) following the manufacturer's instruction. RNA was reverse transcribed into cDNA by using the Taqman Reverse Transcription Reagents kit (Applied Biosystems) with random hexamers. Human LIF, VEGF, HIF-1α, HIF-2α and actin mRNA levels were determined in Step-one Plus real-time PCR System (Applied Biosystems). All primers were purchased from Applied Biosystems. Real-time PCR was done in triplicate with TaqMan PCR mixture (Applied Biosystems). The expression of genes was normalized to the actin gene.

### Western-blot assays

Standard Western-blot assays were used to analyze the levels of protein. Antibodies against HIF-1α (sc-10790, Santa Cruz Biotechnology, 1:500), anti-HIF-2α (ab199, Abcam, 1:1500), anti-LIF (AF-250-NA, R&D, 1:500 dilution) and anti–β-actin (A5441, Sigma) antibodies were used in this study.

### Measurement of LIF production by ELISA assays

Cells cultured under the hypoxic and normoxic conditions were lysed in cell extraction buffer for ELISA assays. The LIF protein levels were measured by using a Quantikine Human LIF immunoassay kit (R&D Systems) in accordance with the manufacturer's instruction.

### Construction of reporter plasmids

The fragments containing the potential HRE sites (5′-G/ACGTG-3′) identified from the *LIF* promoter (A: 582bp~-578bp; B: 1123bp~-1119bp; C: 1769bp~-1765bp; D: 2791bp~2787bp) were amplified by following PCR primers. For HRE-A, forward primer: 5′ CGG GGT ACC AGC ACC CGG AGG AGG AAA CTG G 3′, reverse primer: 5′ CCG CTC GAG CGG GGC CGG CGT GGA CTT 3′; for HRE-B, forward primer: 5′ CGG GGT ACC GGC TGG GCA GGT CTG GGA TGT GG 3′, reverse primer: 5′ CCG CTC GAG GTG GGG AGC CGG GCA AAA TGA GC 3′; for HRE-C, forward primer: 5′ CGG GGT ACC CGG GCG ACG GGG GTT TG 3′, reverse primer: 5′ CCG CTC GAG TTA CTG CTC CTA TAC ACT TGC TCT GGG G 3′; for HRE-D, forward primer: 5′ CGG GGT ACC GCC ATG CCC TTC GCC CTC TCA 3′, reverse primer: 5′ CCG CTC GAG TGC CAT CCA ACC CAT CAC TGC TCT 3′. A HRE site in the *VEGF* promoter (from −1080bp to −874bp upstream of transcription initiation site) was amplified using following primer set: forward: 5′-CGG GGT ACC CCT CAG TTC CCT GGC AAC ATC TG-3′, reverse: 5′-CCG CTC GAG GAA GAA TTT GGC ACC AAG TTT GT-3′. Each of forward primers and reverse primers contains a KpnI site and an XhoI site, respectively. These PCR fragments were cloned into pGL2-Basic Luciferase Reporter Vector (Promega) at KpnI-XhoI restriction sites. All constructs were confirmed by DNA sequencing.

### Luciferase activity assay

To study whether hypoxia transactivates the pGL2-reporter plasmids described above, cells were transiently transfected with the pGL2-reporter plasmids containing one copy of each potential HRE sites together with pRL-null (Promega) as an internal control to normalize transfection by using Lipofectamine 2000 (Invitrogen). Cells were then subjected to hypoxia treatment for 36 h. To study the transactivation activity of HIF-2α on pGL2-reporter plasmids, cells were co-transfected with pGL2-reporter plasmids together with HIF-2α expression plasmids. The luciferase activity was measured by using the Luciferase assay kit (Promega) and normalized with the internal standard.

### ChIP assays

ChIP assays were performed by using a ChIP assay kit (Millipore) in accordance with the instructions of the manufacturer. In brief, cells cultured under the hypoxic or normaxic conditions or transfected with HIF-2α expression plasmids were subjected to ChIP assays with anti-HIF-1α or anti-HIF-2α antibodies. Normal IgG was used as a control for nonspecific binding of genomic DNA. DNA fragments pulled-down by antibodies were recovered and subjected to real-time PCR and conventional PCR by using the PCR primer sets described above with the omission of restriction enzyme recognition sequence.

### IHC staining assays

High-density human colorectal carcinoma tissue microarrays (CO6161) containing duplicated cores for 293 cases of colorectal cancers were purchased from US Biomax. Nine cases with poor core quality were excluded for analysis. IHC staining for LIF and HIF-2α was performed as previously described [17]. In brief, tissue sections were deparaffinized in xylene and rehydrated with ethanol. After pre-incubation with 10% normal goat serum in PBS (pH 7.5), tissue sections were incubated with primary antibodies, including anti-LIF (AF-250-NA, R&D, 1:50 dilution) and anti-HIF-2α antibodies (ab199, Abcam, 1:100 dilution), for overnight at 4°C. Tissue sections were then stained with biotinylated secondary antibody (Vector). Immunoreactivity was detected by using a Vectastain Elite ABC kit (Vector). The IHC results were scored according to the percentage of cells showing positive staining: -: 0% to < 10%; +: > 10%. Two cases of colorectal cancer specimens with positive staining of LIF and HIF-2α and two cases of normal adjacent specimens with negative staining of LIF and HIF-2α were included as positive and negative controls, respectively.

### Statistical analysis

The data were expressed as mean ± SD. The association between HIF-2α expression levels and LIF expression levels were analyzed by Fisher exact test. All other *p* values were obtained by using two-tailed Student's *t*-test. Values of *p* < 0.05 were considered to be significant.
